# Selenium intake help prevent age-related cataract formation: Evidence from NHANES 2001–2008

**DOI:** 10.3389/fnut.2023.1042893

**Published:** 2023-01-27

**Authors:** Baiwei Xu, Zhongwei Liu, Jiangyue Zhao, Ziyan Yu

**Affiliations:** ^1^Department of Ophthalmology, The Fourth Affiliated Hospital of China Medical University, Shenyang, China; ^2^Department of Ophthalmology, Eye Hospital of China Medical University, Shenyang, China; ^3^Key Lens Research Laboratory of Liaoning Province, Shenyang, China

**Keywords:** cataract, trace elements, National Health and Nutrition Examination Survey (NHANES), selenium, cross-sectional study

## Abstract

**Introduction:**

Cataract is one of the leading causes of blindness and visual impairment, about 16 million people around the world. Trace elements play an important role in a variety of the processes in human body. This study aimed to investigate the association between daily dietary intake of trace elements and age-related cataract incidence based on data from the National Health and Nutrition Examination Survey (NHANES) 2001–2008.

**Methods:**

Iron, zinc, copper, and selenium were conducted in this study among subjects aged 50 years and older for African Americans and 55 and older in US adults. Multivariate logistic regression analysis was used in different models to investigate the association of trace elements intake and cataract.

**Results:**

After screening, 7,525 subjects were ultimately included in this study. A significant negative association was found between selenium intake and cataract incidence in adjusted models using multivariate logistic regression analysis (model 1: OR = 0.998, 95% CI = 0.997–1.000; model 2: OR = 0.997, 95% CI = 0.995–1.000; and model 3: OR = 0.998, 95% CI = 0.995–1.000). After dividing selenium intake into quintiles, significant negative associations between selenium intake and cataract were observed in the first quintile of model 3, the fourth and fifth quintiles of all models. In subgroup analyses adjusted for age and sex, a significant negative association was observed only in women aged 65–74 years.

**Discussion:**

Our study points out that maintaining daily dietary selenium intake at higher levels is helpful for cataract prevention, and that increasing daily dietary selenium intake in American women aged 65–74 years may contribute to the prevention of age-related cataract. The intakes of iron, zinc, copper may not be associated with age-related cataract.

## 1. Introduction

Cataract is usually defined as opacification of the lens. Cataract is one of the leading causes of blindness and visual impairment, about 16 million people around the world (1–5). More than 541,000 cataract extraction procedures are performed at a cost of more than $3.8 billion each year in the United States (6). This indicates that cataract has a serious burden on human health and socioeconomy. Age related cataract is usually defined as cataract occurring at age 50 and older (7). Oxidative stress mechanisms is considered to have a part in the pathological process of cataract formation: when oxidative damage in the lens continuously accumulates until its intrinsic antioxidant capacity is exceeded, it will lead to the aggregation of lens proteins and apoptosis of human lens epithelial cells (8, 9). Epidemiological studies have revealed several risk factors for age related cataract, such as age, obesity, diabetes, smoking, and low socioeconomic status (10–14).

Currently cataract treatment modalities for which efficacy has been affirmed are surgery only, and the most commonly used surgical strategy is phacoemulsification and lens replacement (15). Small incision cataract surgery has been widely used, which accelerates the post-operative recovery of patients and improves the quality of post-operative vision (4). However, because the complications associated with cataract surgery similarly affect the visual quality of patients, such as posterior capsular disruption, retinal detachment, progressive myopic traction maculopathy, etc. (16–18). Therefore, effective prevention of cataractogenesis is perhaps the best way to combat the visual damage brought about by cataracts.

Trace elements in the human body include copper, selenium, zinc, manganese, cobalt, chromium, and molybdenum, among others, which function as co-factors or as prosthetic groups located in the enzymes (19–23). The role of these trace elements in diseases is the focus of current research. Currently, researchers have observed that differences in trace element levels exist in the aqueous humor, lens, plasma of cataract patients (24, 25). This difference is similarly present in other age-related eye diseases (26). Shearer et al. found that only selenium was able to cause cataract alone, and that seven other trace elements prevented cataract induced by selenium, with mercury showing the strongest protective ability (27). Selenite also has a strong ability to induce cataract, and because of this property, a rat model of selenite induced cataract has been widely used in cataract related research (28–30). These studies were all able to indicate the close relationship between trace elements and cataract. However, recently Post et al. found that low serum selenium levels maybe a risk factor of age-related cataract (31). This is in contrast to previous findings, perhaps indicating that the effect of selenium on cataract remains to be explored in a deeper step. The effects of other trace elements on cataracts and the specific molecular mechanism are also urgent to be confirmed by further studies.

To further explore the relationship between trace elements and cataract, we carried out this study. This study used National Health and Nutrition Examination Survey (NHANES) database and aimed to analyze the association between trace element intake and incidence of cataract, which may provide a foundation for guiding trace element supplementation.

## 2. Materials and methods

### 2.1. Data source and subjects selection

This study is based on NHANES data from 2001 to 2008. NHANES is a national cross-sectional research program aimed to assess the physical status of ordinary Americans, performed by the National Center for Health Statistics (NCHS). The NHANES subjects were all U.S. non-institutionalized civilian participants, all of whom had accepted comprehensive measurements. Each cycle of NHANES is an independent cross-sectional survey. The NCHS research ethics review board approved the survey protocol for NHANES. All participants gave written informed consent (32).

Referred to previous studies, we set the inclusion criteria for age as 50 years or older for non-Hispanic blacks and 55 years or older for other races (33). In the study, a total of 28,332 subjects who underwent ophthalmic examination were identified, and 7,525 subjects were finally included. 20,807 subjects were excluded for the following reasons: (1) No valid data on cataract diagnosis; (2) No valid data on trace element intake; (3) Not meeting the above age inclusion criteria.

### 2.2. Cataract identification criteria

National Health and Nutrition Examination Survey asked participants aged 20 years and older if they had undergone ophthalmic surgery for cataracts prior to their ocular examination (34). If participants answered yes, they were defined as having cataract in this study. Participants with non-response or uncertain response were excluded. Because of the increased rate and lower threshold for cataract surgery in the United States (4, 35), self-reported cataract surgery may be able to represent clinically meaningful cataract. This defining criterion for cataract has also been used in previous studies (36, 37).

### 2.3. Determination of intake of various trace elements and daily energy intake

Dietary data were collected in the in-person interview using the Automated Multiple Pass Method (AMPM). Participants recalled all of the foods they had consumed on the previous day and told staff. The staff calculated the amount of various nutrients they ingested daily based on what the subjects said. The AMPM is a USDA′s dietary data collection instrument and a fully computerized recall method. The NHANES Mobile Examination Center (MEC) provided a set of measuring guides that facilitated participants to describe the amount of foods they had ingested (38).

Four trace elements of iron, copper, zinc, selenium were included in our study. Because in the NHANES dietary survey database, there are no dietary intake data for other trace elements except these four. Daily energy intake was used to represent the total amount of food the participants consumed on a daily basis.

### 2.4. Covariates assessment

We selected demographic variables such as age, race/ethnicity, sex, and education level to be included as covariates in this study. These demographic data were obtained through computer-assisted face-to-face interviews (39). Social status and living status affect physical wellbeing. But these indicators could not be quantified, so we took the above demographic data to evaluate the social status and living status of participants.

Diabetes mellitus, smoking, obesity/overweight are all risk factors for age-related cataract, therefore (40–44), they were also included as covariates in this study. Diabetes status was defined by self-reported diagnosis (45). Smoking status was defined by serum cotinine levels to reflect both direct and indirect smoking quantity (46, 47). Obesity/overweight was reflected by body mass index (BMI). BMI was calculated by the weight in kilograms divided by the square of height in meters (kg/m^2^) (48).

### 2.5. Statistical analysis

All statistical analyses were performed using SAS 9.4. NHANES uses a stratified, multistage sampling method, so we incorporated sampling weights and strata, sampling units in our statistical analysis to account for the complex sampling design. For continuous variables, we used means and standard errors (SE) expressed with *t*-test to compare participants′ characteristic variables. For categorical variables, we expressed percentages and SE with the Rao Scott Chi-square test to compare participants′ characteristic variables. Logistic regression models were used to determine the association of various trace element intakes with the presence of cataracts. To better determine their association, we selected three models. Model 1 was adjusted by age, race, gender, and education level to correct the influence of demographic characteristics. Model 2 = Model 1 and adjusted by diabetes, BMI and daily energy intake to correct the influence of daily food intake, obesity, and diabetes. Model 3 = Model 2 and adjusted by serum cotinine to correct the influence of smoking. Since a significant association between selenium and cataract was observed, to better investigate the association between the two, we further performed quintile regression between selenium and cataract after dividing the intake levels of selenium into quintiles. Finally, because age and sex were the most prominent risk factors, we performed subgroup analyses for age and sex. Because of the setting of the inclusion criteria, participants 50–54 years of age were all black, and to avoid this selection bias, subgroup analyses were performed starting at age 55 in each decade as one group. Gender was grouped in males and females. All statistical analysis results with a two-tailed *p* value < 0.05 were considered significant.

## 3. Results

### 3.1. Description of baseline information of the study sample

Table 1 shows the demographic data as well as other characteristic data of the participants with and without cataracts. Of all included participants, 1,570 had cataracts, 18.10% of the total after weighting, 5,955 did not have cataracts, and 81.90% of the total after weighting. Participants with cataracts all had significantly lower intakes of trace elements, including iron (13.948 vs. 14.812 mg), zinc (10.269 vs. 11.114 mg), copper (1.151 vs. 1.261 mg), selenium (85.698 vs. 98.267 μg). Significant differences were also observed in other covariates. Older age, female sex, non-Hispanic white race, and lower educational level groups were all more likely to have cataracts.

**TABLE 1:** Baseline information for the study sample.

Variables		Cataract (+)	Cataract (–)	*p*-value
**Continuous variables, mean (SD)**				
Unweighted counts		1570	5955	
Age (years)		75.040 (0.288)	64.840 (0.167)	<0.001
Iron intake (mg)		13.948 (0.235)	14.812 (0.193)	<0.001
Zinc intake (mg)		10.269 (0.214)	11.114 (0.241)	0.001
Copper intake (mg)		1.151 (0.0308)	1.261 (0.0220)	<0.001
Selenium intake (μg)		85.698 (1.309)	98.267 (1.284)	<0.001
Energy (kcal)		1672.820 (19.086)	1886.280 (20.793)	<0.001
BMI (kg/m^2^)		28.051 (0.190)	28.862 (0.118)	<0.001
Serum cotinine (ng/mL)		28.932 (2.518)	50.664 (2.167)	<0.001
**Category variables, (%)**				
Cataract		18.100 (0.700)	81.900 (0.700)	
Gender	Male	35.800 (1.200)	46.800 (0.700)	<0.001
	Female	64.200 (1.200)	53.200 (0.700)	
Race	Mexican American	2.700 (0.500)	4.100 (0.600)	<0.001
	Other Hispanic	2.300 (0.600)	2.700 (0.600)	
	Non-Hispanic white	84.900 (1.500)	76.600 (1.800)	
	Non-Hispanic black	6.600 (0.800)	12.800 (1.300)	
	Other race–including multi-racial	3.600 (0.600)	3.700 (0.500)	
Education level	Less than 9^th^ grade	16.000 (1.200)	9.000 (0.700)	<0.001
	9–11^th^ grade (Includes 12^th^ grade with no diploma)	16.300 (1.400)	12.300 (0.700)	
	High school grad/GED or equivalent	26.700 (1.700)	27.700 (1.000)	
	Some college or AA degree	24.200 (1.300)	25.000 (1.000)	
	College graduate or above	16.800 (1.600)	26.000 (1.400)	
Diabetes mellitus	(+)	27.000 (1.500)	16.200 (0.800)	<0.001
	(–)	73.000 (1.500)	83.800 (0.800)	

### 3.2. Association between the intake of iron, zinc, copper, selenium, and the presence of cataract

Table 2 shows the associations that existed between the intake of various trace elements and cataract as addressed by multivariate logistic regression models. A significant negative association between selenium intake and incident cataract was shown in all models (model 1: OR = 0.998, 95% CI = 0.997–1.000; model 2: OR = 0.997, 95% CI = 0.995–1.000; model 3: OR = 0.998, 95% CI = 0.995–1.000). No significant association with cataract was observed for the intakes of iron, zinc, copper.

**TABLE 2:** Association between intake of iron, zinc, copper, selenium and cataract.

Variables	Model 1^a^ OR (95% CI)	*p*-value	Model 2^b^ OR (95% CI)	*p*-value	Model 3^c^ OR (95% CI)	*p*-value
Iron intake	0.999 (0.986∼1.0130)	0.901	0.993 (0.978∼1.00900)	0.370	0.994 (0.978∼1.00900)	0.436
Zinc intake	1.00800 (0.996∼1.0200)	0.179	1.00900 (0.997∼1.0210)	0.138	1.00900 (0.997∼1.0210)	0.154
Copper intake	0.996 (0.937∼1.0590)	0.897	0.987 (0.927∼1.0520)	0.695	0.984 (0.920∼1.0520)	0.625
Selenium intake	0.998 (0.997∼1.000)	0.0481	0.997 (0.995∼1.000)	0.0184	0.998 (0.995∼1.000)	0.0275

^a^Model 1: adjusted for age, race, gender, educational level.

^b^Model 2:further adjusted for diabetes mellitus, BMI, daily energy intake.

^c^Model 3: further adjusted for serum cotinine.

### 3.3. Relationship of different quintiles of selenium with the presence of cataract

Table 3 demonstrates the analysis of the association of different grades of selenium intake with cataract after dividing selenium intake into quintiles. The quintiles of selenium intake levels were 55.9, 75.9, 97.4, and 129.6 μg. In the first quintile of model 3 (OR = 0.985, 95% CI = 0.971–0.999), and the fourth (model 1: OR = 0.979, 95% CI = 0.960–0.998; model 2: OR = 0.979, 95% CI = 0.958–1.000; model 3: OR = 0.977, 95% CI = 0.956–0.998) and fifth quintiles (model 1: OR = 0.996, 95% CI = 0.992–0.999; model 2: OR = 0.996, 95% CI = 0.992–1.000; model 3: OR = 0.996, 95% CI = 0.992–0.999) of all models, we observed a significant negative association between selenium intake and cataract.

**TABLE 3:** Association between selenium intake levels and cataract in different quintiles.

Variables		Model 1^a^ OR (95% CI)	*p*-value	Model 2^b^ OR (95% CI)	*p*-value	Model 3^c^ OR (95% CI)	*p*-value
Selenium intake	Q1 (<55.9 μg)	0.995 (0.980∼1.010)	0.512	0.988 (0.974∼1.002)	0.0815	0.985 (0.971∼0.999)	0.0422
	Q2 (55.9∼75.9 μg)	0.992 (0.957∼1.028)	0.647	0.987 (0.949∼1.0280)	0.527	0.985 (0.945∼1.0280)	0.490
	Q3 (75.9∼97.4 μg)	0.993 (0.963∼1.0250)	0.678	0.990 (0.961∼1.0190)	0.477	0.990 (0.960∼1.0200)	0.486
	Q4 (97.4∼129.6 μg)	0.979 (0.960∼0.998)	0.0318	0.979 (0.958∼1.000)	0.452	0.977 (0.956∼0.998)	0.0343
	Q5 (>129.6 μg)	0.996 (0.992∼0.999)	0.0172	0.996 (0.992∼1.000)	0.0305	0.996 (0.992∼0.999)	0.0273

^a^Model 1: adjusted for age, race, gender, educational level.

^b^Model 2:further adjusted for diabetes mellitus, BMI, daily energy intake.

^c^Model 3: further adjusted for serum cotinine.

### 3.4. Subgroup analyses for age and sex

Table 4 presents the association of cataracts and selenium in male and female participants at different ages. In accordance with our results, we only observed a significant negative association in women aged 65–74 years in all models (model 1: OR = 0.994, 95% CI = 0.990–0.999; model 2: OR = 0.992, 95% CI = 0.985–1.000; model 3: OR = 0.992, 95% CI = 0.984–1.000). No significant association was observed at other ages for women and at all ages for men.

**TABLE 4:** Association between selenium intake and cataract in different ages and sex.

Variables		Model 1^a^ OR (95% CI)	*p*-value	Model 2^b^ OR (95% CI)	*p*-value	Model 3^c^ OR (95% CI)	*p*-value
Male	55∼64 years	0.995 (0.988∼1.00300)	0.191	0.998 (0.985∼1.0110)	0.763	0.998 (0.985∼1.0120)	0.809
	65∼74 years	1.000 (0.996∼1.00400)	0.968	1.00300 (0.998∼1.00800)	0.189	1.00300 (0.998∼1.00700)	0.219
	75∼85 years	1.000 (0.997∼1.00300)	0.902	0.998 (0.994∼1.00200)	0.255	0.998 (0.994∼1.00200)	0.307
Female	55∼64 years	1.003 (0.997∼1.00800)	0.312	0.990 (0.961∼1.0190)	0.943	1.00100 (0.994∼1.00800)	0.822
	65∼74 years	0.994 (0.990∼0.999)	0.0235	0.992 (0.985∼1.000)	0.0433	0.992 (0.984∼1.000)	0.0387
	75∼85 years	0.997 (0.993∼1.00200)	0.233	0.996 (0.991∼1.00100)	0.120	0.996 (0.991∼1.00100)	0.141

^a^Model 1: adjusted for race, educational level.

^b^Model 2:further adjusted for diabetes mellitus, BMI, daily energy intake.

^c^Model 3: further adjusted for serum cotinine.

## 4. Discussion

Our study included large-scale cross-sectional data from four NHANES cycles. Logistic regression results showed a significant negative association between selenium intake and cataract. There was no significant associations between the intake of iron, copper, and zinc and cataract. Therefore, our study points out that increasing selenium intake in daily diet may decrease the risk of cataract. This notion was subsequently confirmed in the multivariable logistic regression model performed after dividing selenium intake into quintiles. Because significant negative associations were observed in the first quintile of model 3, the fourth, and fifth quintiles of all models, we hypothesized that maintaining daily dietary selenium intake at lower or higher levels is helpful for cataract prevention. In subgroup analyses adjusted for age and sex, the inverse association of selenium intake with cataract was observed only among US women 65–74 years of age. Four trace elements, iron, zinc, copper, and selenium, have a non-negligible role in the human body and they are involved in all aspects of human physiological activities (49–54), as well as in the visual system (55–57). Oxidative stress mechanism played an important role in the pathological process of cataract, in which iron, zinc, copper were involved (58–64). However, the relationship between these three trace elements and cataract could not be well confirmed in the current study (62, 65–71). This is consistent with our study, which perhaps indicated that the intake of these three trace elements had a negligible relationship with cataract.

In our results, selenium was the only significant variable, which deserves our high attention. All the time, rats with high-dose selenite intake have frequently been used to prepare animal models of cataract, which is able to laterally reflect the promoting effect of high-dose selenium on cataract (28, 72). Post et al.’s cross-sectional findings showed that low-serum selenium showed a positive association with age-related cataract only in the first quartile range of serum selenium levels (OR = 7.969, *p* < 0.01) (31). While the results of the SELECT Eye Endpoints (SEE) study conducted by Christen et al. indicated that an additional 200 μg/d of L-selenomethionine daily as a supplement source of selenium during an average 5.6 years of follow-up failed to observe a protect effect of selenium on age-related cataract (33). In the study of Xiangjia Zhu et al., after oral supplementation of different doses of selenium to rats with cataract induced by naphthalene solution, the slowing of the increase of lens density or the decrease of turbid density could be observed at all selenium intake doses. And they also observed an elevation of glutathione peroxidase (GPx) activity in the lens of Se supplemented group rats. This suggests that selenium supplementation is able to slow down the development of naphthalene induced cataract by slowing down oxidative stress (73). These above results suggest that the effect of selenium on cataract remains inconclusive, which is still a topic of investigation. Dietary intake is the main body′s access to selenium and selenium in food includes both organic and inorganic forms. The organic forms of selenium include selenomethionine and selenocysteine, and their bioavailability is high, up to 90–95%; the inorganic form of selenium includes selenite, selenides, etc., and its bioavailability is low, only 80–85% (54). The effects of these two forms of selenium on cataract are distinct. For the organic form of selenium, after the tRNA mediated incorporation of selenocysteine, these selenium can synthesize a variety of selenoproteins, including GPx and thioredoxin reductases (TrxR), among others, which have a powerful antioxidant capacity; organic forms of selenium or are able to prevent cataractogenesis by combating oxidative stress (54, 74–76). The inorganic form of selenium is in the oxidation state; this might aggravate oxidative stress and thus promote cataract (74). In addition, selenite is also able to promote cataracts through mechanisms such as altered epithelial metabolism, calcium accumulation, calpain induced proteolysis, crystallin precipitation, phase transition, and cytoskeletal loss (28). Notably, Huang et al. also found that the effect of selenite on the lens changed over time, with a 30% decrease in DNA replication in lens epithelial cells observed at 6–12 h after administration of the selenite into rats, but an 80% increase in DNA replication in lens epithelial cells was observed by 24 h. This suggests that selenite, after a period of action on the lens, has an effect that shifts from damage caused by oxidative stress to repair of the lens epithelium (77). Combined with our results, we speculate that the reason that selenium only has a protective effect on cataract at lower and higher doses may be the gap in bioavailability between the organic and inorganic forms of selenium and the different maintenance times of high concentrations of selenite within the lens resulting in different effects. Because the bioavailability of the organic form of selenium is greater than that of the inorganic form, at low dietary doses of selenium, the organic form of selenium absorbed by the body predominates, at which point the organic form of selenium exerts antioxidant effects through a series of biological metabolism, thereby playing a protective role against cataracts. But when selenium intake increases, the uptake of inorganic forms of selenium increases in the body, and a range of damaging effects on the lens begin to manifest. Since at this time the protective effect exerted by the organic form of selenium was similarly enhanced with increasing dose, it did not show an absolute cataract promoting negative effect. However, as selenium intake continues to rise to higher levels, it is sufficient within the lens to maintain higher selenite concentrations for an extended period of time at which point the reparative effects of selenite on lens cells begin to manifest and, together with selenoproteins, exert a protective effect on the lens, thereby against cataracts. The reason why selenium gradually exerted different effects on cataract at different intake levels is because the main aggregation sites of selenium in the human body are liver, muscle, kidney. Of the selenium ingested by humans, the dose of selenium able to aggregate into lens is low. Thus, only when a large change in selenium intake occurs can it lead to an influential change in the amount of selenium accumulated within the lens, resulting in a potent effect on the lens (78). However, although we found a protective role of selenium in cataract, because selenium is an essential nutrient for humans, prevention of cataract by reducing selenium intake seems an unwise regimen. Therefore, we more recommend that elderly people prevent cataract by moderately increasing their intake of selenium in their daily diet.

In the results of subgroup analysis, no significant association was observed in males of all ages. This is similar to the findings of Christen et al. (33). A significant negative association was observed in women aged 65–74 years, suggesting that increased selenium intake may be a protective factor for women in this age group of cataract. This result is presented, perhaps because of the role that estrogen withdrawal plays in cataracts (79). Another NHANES study showed that the mean age at menopause among US women enrolled in the study from 2001 to 2008 was 49.9 years (80). The course of cataracts is long, ranging from the onset of pathological changes to patients′ self-conscious symptoms for up to several years. And when visual symptoms occur, the time that patients go to the hospital and undergo cataract surgery is delayed because of personal psychological or socioeconomic factors. The results observed in our study should therefore be broadly consistent with the actual situation. Taken together, for women, taking selenium supplements at the beginning of menopause may help prevent cataract.

In the present study, since no direct data were available on the prevalence of cataracts in NHANES 2001–2008, we can only roughly estimate the prevalence of cataracts by cataract surgery status. This approach has been widely used in previous cataract studies using the NHANES database (36, 37, 81, 82). However, the plausibility of this approach has not been discussed in detail in previous studies, but we believe it is necessary. First, the study by Varma et al. estimated that the prevalence of cataract was 19.50% in the US population (83), and the meta-analysis by Hashemi et al. indicated that the worldwide cataract prevalence was approximately 17.20% (84). While in the present study, cataract surgery accounted for 18.10% of all participants, which was consistent with the above data. Second, in terms of cataract surgical coverage (CSC), CSC was defined as the percentage of cataract patients who underwent cataract surgery as a percentage of the total number of cataract patients (85). To the best of our knowledge, there are no studies conducted on CSCs for the US population, but based on the Rapid Assessment of Avoidable Blindness (RAAB) survey data, researchers have conducted extensive studies on CSCs for other countries, for example, Tabin et al.’s study indicated that CSCs can reach 50–70% in most developing countries (85), while Szabó et al. indicated that CSCs in Hungary, which is the same developed country as the US, are even higher up to 90% (86). Based on the above data, we can predict that the United States, as a developed country and with a high level of medical care, should also have CSCs at a high level, and most cataract patients are able to undergo cataract surgery. Finally the reduced cost of cataract surgery and the higher health gains for patients after surgery are also reasons for increased CSCs (87). Taken together, we believe that the cataract surgery data in this paper are broadly representative of the prevalence of cataract.

Although our study benefited from a reasonable sampling design to obtain a large sample size and nationally representative population, there are still certain limitations of this study that deserve to be explored. First, according to the NHANES method of ophthalmological examination, we can only know patients who have had cataract surgery and give a rough estimate of cataract occurrence in terms of cataract surgery status. The inability to detect those patients who had cataracts but remained unoperated would therefore make us underestimate the incidence of cataracts in our study. Second, cataracts are divided into subtypes, each of which has some differences in pathogenesis and risk factors that are not available in NHANES ophthalmological examination results. Third, dietary nutrient intake data were verbally ascertained through participants′ recall, and although based on the NHANES complete and rigorous measurement method, which enables accurate access to dietary nutrient data for a large proportion of participants, there may still be a small proportion of participants with varying degrees of recall bias. Fourth, because the contents of these trace elements in serum, aqueous humor, lens were not present in the results of the 2001–2008 NHANES study, these findings may be affected by other factors, such as different levels of subject digestive absorption, which could not be eliminated by adjustment. Finally, as a cross-sectional study, causality between observed variables cannot be directly concluded. However, our study also has a number of irreplaceable strengths. First, this is the first study to investigate the association between trace element intake and the risk of incident cataract through a nationally representative population-based survey. Second, to the best of our knowledge, this is the first study to report that high and low selenium intakes are protective against cataracts. Third, this is also the first study to report differential effects of selenium intake on cataracts in different genders. Fourth, it is also the first study that has explored the availability of data from cataract related studies in NHANES 2001–2008 in detail.

## 5. Conclusion

Our study points out that maintaining daily dietary selenium intake at higher levels is helpful for cataract prevention, and that increasing daily dietary selenium intake in American women aged 65–74 years may contribute to the prevention of age-related cataract. The intakes of iron, zinc, copper may not be associated with the incidence of age-related cataract.

## Data availability statement

Publicly available datasets were analyzed in this study. This data can be found here: https://www.cdc.gov/nchs/nhanes/index.htm.

## Ethics statement

Approval for data collection was obtained from the NCHS Research Institutional/Ethics Review Board (IRB/ERB) (Protocol #98-12, Protocol #2005-06, Continuation of Protocol #2005-06). The patients/participants provided their written informed consent to participate in this study.

## Author contributions

BX: conceptualization, software, investigation, resources, and data curation. BX and JZ: methodology. ZY and JZ: supervision and project administration. BX, ZY, and JZ: writing—original draft preparation and writing—review and editing. ZY: funding acquisition. BX and ZL: formal analysis. ZY and ZL: validation. All authors have read and agreed to the published version of the manuscript.
